# LncRNA FTX accelerates the progression of hepatocellular carcinoma by FTX/miR-374a-3p/HMGB1 pathway

**DOI:** 10.7150/ijms.106867

**Published:** 2025-02-18

**Authors:** Min Zhang, Songman Yu, Shang Gao, Haiyan Bu, Lihua Duan, Yan Huang

**Affiliations:** 1 Department of Infectious Diseases, Hunan Key Laboratory of Viral Hepatitis, Xiangya Hospital, Central South University, Changsha, China.; 2 National Clinical Research Centre for Geriatric Disorders, Xiangya Hospital, Central South University, Changsha, China.

**Keywords:** Lnc-FTX, hepatocellular carcinoma, RBMX, HMGB1, miR-374a-3p

## Abstract

The situation regarding Hepatocellular carcinoma (HCC) is severe, with high incidence and mortality rates worldwide. Abnormal expression of long noncoding RNAs (LncRNAs) has been implicated in the progression of malignant tumors. Although there are reports on LncRNA FTX (Lnc-FTX) in HCC, the findings are still contradictory, leaving its role unclear. This research aims to examine the relationship between Lnc-FTX expression and clinical prognosis in HCC, assess its impact on HCC cell biological functions, and elucidate the underlying mechanisms involved. Our findings demonstrate that patients in the high-expression group exhibit more severe TNM staging and poorer overall survival (OS) rates based on data from HCC patients in cohort 1 (n=27). Lnc-FTX expression is upregulated according to both the GSE 77314 and TCGA databases. This suggests that Lnc-FTX may serve as a potential prognostic biomarker for HCC. Overexpressed level of Lnc-FTX promotes proliferation, migration, and invasion while reducing the apoptotic rate in Hep3B and MHCC-LM3 cells. Conversely, the knockdown of Lnc-FTX yields opposite effects. RNA pulldown assay and mass spectrometry reveal that Lnc-FTX combines with RNA binding motif protein X-linked (RBMX), which in turn enhances the RNA stability of Lnc-FTX. Additionally, Lnc-FTX can sponge miR-374a-3p, thereby targeting High mobility group box 1 protein (HMGB1). In summary, high expression of Lnc-FTX possesses clinical value in predicting poor prognosis in HCC. As an oncogene, it promotes the malignant functions of HCC through the RBMX/Lnc-FTX interaction and the Lnc-FTX/miR-374a-3p/HMGB1 signaling pathway.

## Introduction

Primary hepatic cancer (PHC) is the sixth most commonly diagnosed cancer, and ranks as the third leading cause of cancer-related deaths globally in 2022, accounting for 866,136 new cases and 758,725 deaths 1,2. HCC, the predominant type of PHC, constitutes about 75%-85% 3. The difficulty in early diagnosis of HCC, along with its high recurrence and metastasis rates, is the main reason for high mortality 4,5. For early-stage HCC patients (stage 0 or stage A), curative treatment options include liver transplantation, surgical resection, and radiofrequency ablation 6. For advanced HCC, systemic therapy is recommended. First-line agents consist of small molecule inhibitors such as Sorafenib and Lenvatinib 7,8, while second-line treatment options include immune checkpoint inhibitors, such as Nivolumab (PD-1) 9. Additionally, epigenetic therapies are under further investigation, including DNA methyltransferase inhibitors (zebularine) and miRNA-targeted therapies (miR-124) 6. Currently, emerging research suggests that LncRNAs play a role in HCC and may serve as novel therapeutic targets. SiHSAL3 nanoparticles loaded with oncogenic lncRNA HSAL3 inhibitors could be a potential therapeutic drug for HCC 10.

LncRNAs are a group of non-coding RNA transcripts (> 200 nt) 11, which are now recognized as epigenetic regulators that play a role in tumor malignant processes, such as proliferation 12, invasion and metastasis 13, as well as metabolism 14. Our team's preliminary research highlights the significant role of lncRNA in HCC. SEMA 6A-AS1 has been identified as a potential prognostic biomarker for HBV-related HCC 15. LncRNA TP73-AS1 functions as an oncogene in HCC by promoting M2 macrophage infiltration via the miR-539/MMP-8/TGF-β1 signaling pathway 16 and regulates HCC cell proliferation through the miR-200a/HMGB1 pathway 17.

Lnc-FTX is derived from the FTX gene, a highly conserved gene, which is located on the human X chromosome q13.2.^18^ Originally discovered as an important regulator of X-chromosome inactivation, it can regulate X-inactive-specific Transcript (XIST) activation and expression. Subsequently, numerous studies have demonstrated up-regulation of Lnc-FTX in diverse cancers, including gastric cancer 19, colorectal cancer 20, and other digestive cancers. The research on Lnc-FTX in HCC remains controversial. In the study by Liu F, *et al.*, Lnc-FTX acts as a tumor suppressor gene in HCC by inhibiting epithelial-mesenchymal transition (EMT) and invasion of HCC cells through the miR-374a-5p/Wnt/β-catenin axis 21 and regulating the M1/M2 polarization of Kupffer cells 22, and inhibits cell proliferation by binding with MCM2 23. However, other studies have shown that Lnc-FTX expression is upregulated in HCC, where it contributes to aerobic glycolysis via the PPARγ pathway 24 and promotes HCC progression through the miR-545/RIG-I/PI3K/Akt axis 25. The abnormal expression of Lnc-FTX exhibits tissue heterogeneity, and the molecular mechanism of tumor regulation needs to be further investigated.

HMGB1 has been extensively reported to exhibit pro-carcinogenic effects 26. It can promote tumor invasion, metastasis 27, and proliferation 28 by interacting with receptors (such as RAGE and TLRs), thereby activating downstream signaling pathways including NF-κB/IL-6 29, caspase-1 30, PI3K/AKT 31 and cyclin D1 32. Additionally, nuclear HMGB1 inhibits apoptosis by regulating heat shock protein β-1 33. This study hypothesizes that HMGB1 is a potential target for FTX in regulating HCC.

In this research, the expression of Lnc-FTX in tumor and adjacent non-tumor liver tissues from HCC patients were examined, and its impact on the malignant phenotype of HCC cells was investigated. The potential molecular mechanisms were explored by focusing on RNA-binding proteins (RBPs) and microRNAs that bind to Lnc-FTX. Our study identifies Lnc-FTX as a potential prognostic biomarker and a therapeutic target, thereby enhancing our understanding of the developmental mechanisms underlying HCC.

## Material and Methods

Additional methodologies are listed in Supplementary Material and Methods Doc S1.

### Tissue samples and public databases

Between 2016 and 2021, 27 paired HCC samples and their respective adjacent non-tumor tissues were obtained from tumor surgeries conducted at the Third Xiangya Hospital (Table S1) (HCC cohort 1). All samples were collected strictly in accordance with standard procedures, and stored at -80°C. FTX transcript level data for 371 HCC cases from the The Cancer Genome Atlas (TCGA) database were obtained online from The University of Alabama at Birmingham Cancer data analysis Portal (UALCAN) and Lnc-FTX expression data for 50 HCC patients were obtained from Gene Expression Omnibus (GEO) database.

### Cell culture

MHCC-LM3, Hep3B, MHCC97H, MHCC97L (HCC cell lines) and HEK-293T (embryonic kidney cell line) cells were cultivated in Dulbecco's Modified Eagle Medium (Gibco, USA). HepG2 cells were cultivated in MEM (Gibco, USA). All the cell lines were obtained from the American Type Culture Collection. L02 (the hepatocyte cell line) was purchased from the Culture Collection of the Chinese Academy of Sciences and was maintained in RPMI 1640 medium (Gibco, USA). All cells were cultured in complete medium (basal medium + 10% Fetal Bovine Serum (Gibco, USA) + 1% penicillin-streptomycin (Gibco, USA)). The cell incubator was set to 5% CO2 and 37 °C.

### RNA pulldown assays

Lnc-FTX RNA was obtained by transcription of the FTX gene template containing the T7 promoter using the TranscriptAid T7 High Yield Transcription Kit (K0441, Thermo, USA). Lnc-FTX RNA pulldown was conducted using the RNA-protein PullDown kit (20164, Thermo, USA). Subsequently, biotin-labelled Lnc-FTX was co-incubated with streptavidin magnetic beads for 30 min (room temperature) and further co-incubated with whole cell lysate for 1 hour at 4°C. Following washes with wash buffer for 3 times, proteins were pulled down by the probe-encapsulated magnetic beads. Finally, the eluted proteins samples were analyzed by nanoLC-MS/MS (BiOTREE, China). We performed Tagged RNA affinity purification (TRAP) assay using the TRAP kit (BersinBio, GuangZhou, China) to detect microRNAs that bind to Lnc-FTX. All the relative primers were detailed in Table S2.

### Dual-luciferase reporter assay

Luciferase reporter plasmids should be prepared in advance. Cells were seeded into the 24-well plates (5×10^4^/well). Then the miR-374a-3p or NC mimics were transfected into the cells. Collected cell lysates were used to detect the activities of Renilla and firefly luciferase (RG027, Beyotime, China). The absorbance value was detected using a PerkinElmer spectrophotometer.

### RNA stability assay

Cells were treated with actinomycin D (sigma, USA) after transfection with shNC or shRNA-RBMX. Cells were collected at 0, 4 and 8h after actinomycin D treatment, followed by RT-PCR analysis to measure the expression level of Lnc-FTX. The half-life (t_1/2_) of Lnc-FTX was determined as the time when the expression of Lnc-FTX decreased to 50% after treatment with actinomycin D. The decay rate of Lnc-FTX was determined by non-linear regression curve fitting.

### Statistical analysis

For categorical variables between the two groups, P values were statistically assessed using the χ^2^ test, while Student's t-test was applied for quantitative data. ANOVA was utilized for comparisons involving more than two groups. Statistical values were analyzed and presented using GraphPad Prism 9.0 (CA, USA). Data with p value < 0.05 was considered as statistically significant.

## Results

### The overexpression of Lnc-FTX is linked to poor prognosis in HCC

**Figure 1:**
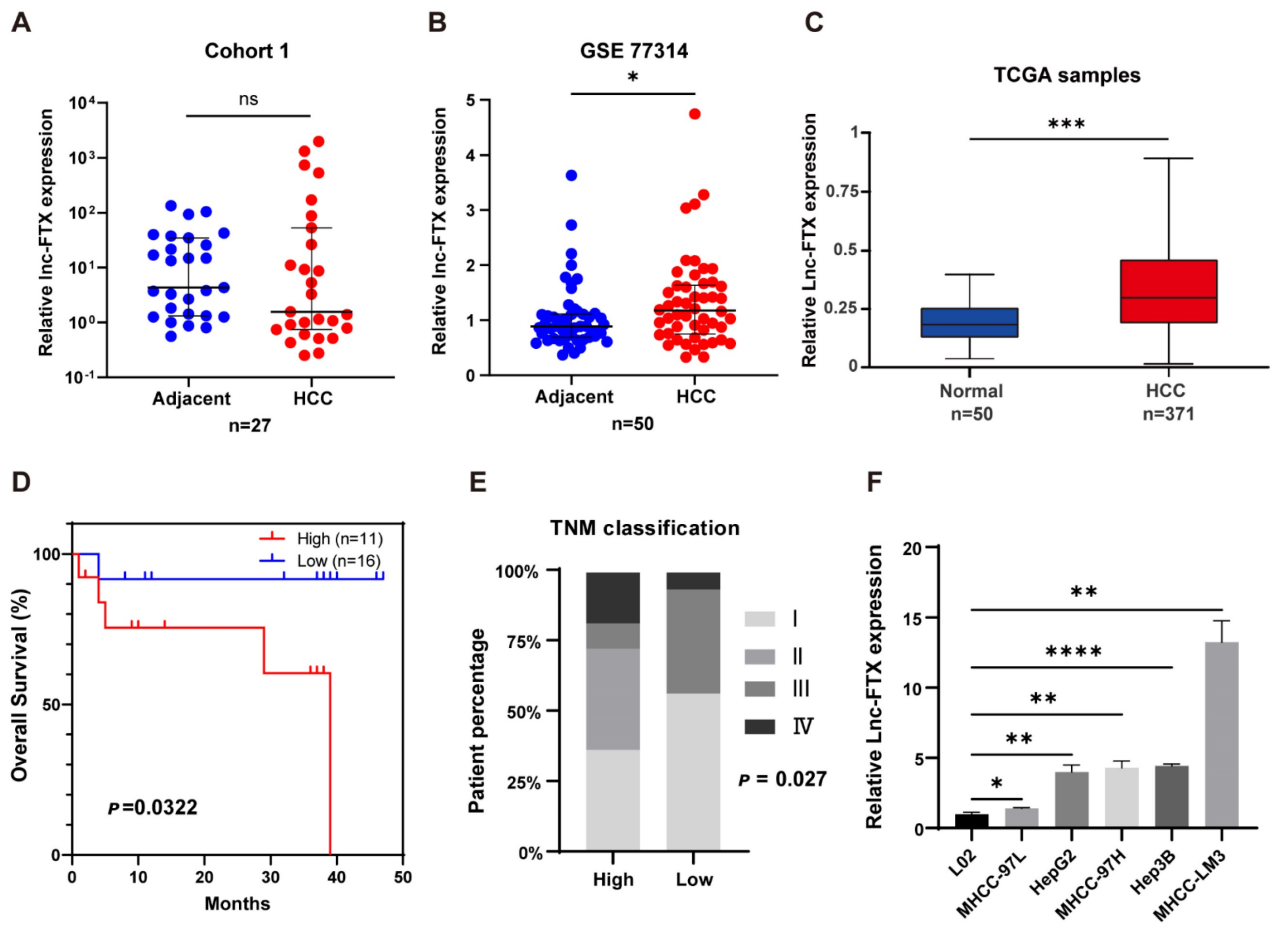
The overexpression of Lnc-FTX is related to the poor clinical prognosis of HCC patients. **(A)** Lnc-FTX levels in 27 pairs HCC and adjacent tissues were measured by RT-PCR;** (B)** Lnc-FTX is significantly upregulated in 50 HCC tissues compared with the matched adjacent normal liver tissues obtained from GEO database GSE77314;** (C)** Lnc-FTX expression level is higer in HCC (n = 371) than in the normal liver tissues (n = 50) obtained from TCGA (UCLCAN);** (D)** Kaplan-Meier survival analysis suggests that HCC patients with higher Lnc-FTX levels had worse overall survival (OS) than those with lower lnc-FTX expression; **(E)** Chi-Squared Test suggests that HCC patients with higher Lnc-FTX levels had higher TNM classification than those with lower Lnc-FTX expression; **(F)** LncRNA FTX expression was detected by RT-PCR in normal human hepatocytes L02 and HCC cell lines, including MHCC-97L, HepG2, MHCC-97H, Hep3B, MHCC-LM3.

To explore the expression of Lnc-FTX in HCC, RT-PCR experiments were performed on tumor tissues and adjacent non-tumor tissues collected from 27 HCC patients. The results showed no significant difference between tumor and adjacent non-tumor tissue. (Figure 1A). Based on data from GEO GSE77314 (Figure 1B) and TCGA (Figure 1C), we observed that Lnc-FTX is significantly upregulated in HCC tissues. Remarkably, the high Lnc-FTX group exhibited significantly poorer OS compared to the low Lnc-FTX group (P<0.05) (Figure 1D). Additionally, clinicopathological analysis showed that patients in high Lnc-FTX group exhibited higher TNM stage (Figure 1E), with detailed data presented in Table 1. Furthermore, expression levels of Lnc-FTX were elevated in HCC cell lines, including MHCC-97L, HepG2, MHCC-97H, Hep3B (Figure 1F). In summary, overexpression of Lnc-FTX was associated with higher TNM stage and poor clinical prognosis.

### Lnc-FTX promotes cell proliferation and inhibits apoptosis in HCC

**Figure 2:**
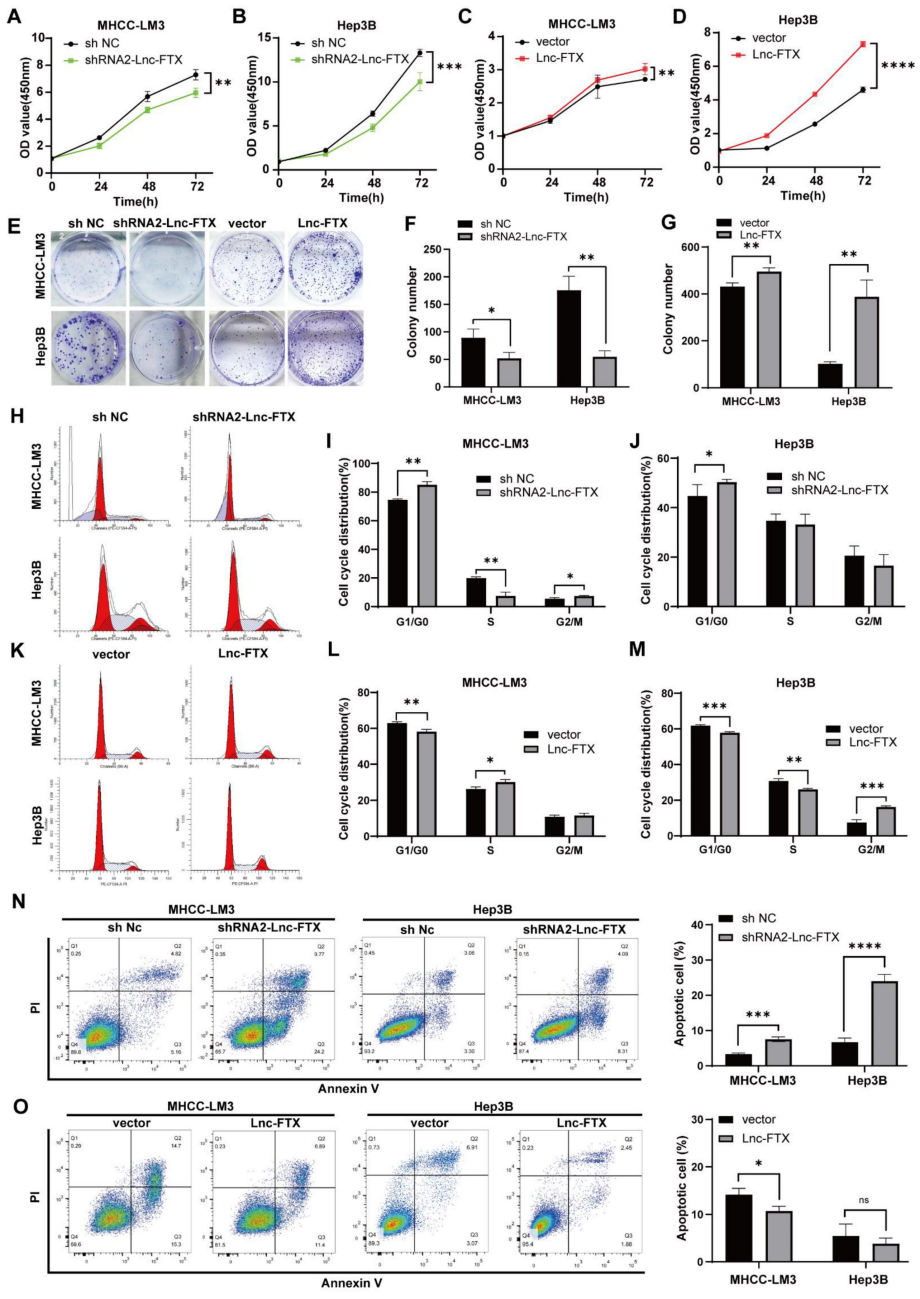
The effect of Lnc-FTX on HCC cell proliferation and apoptosis. **(A-D)** CCK8 assay was performed to measure the cell viability;**(E-G)** MHCC-LM3 cells and Hep3B cells, transfected with shRNA2-Lnc-FTX/shNC and vector/vector-Lnc-FTX, were cultured for 2 weeks to allow colony formation; **(H-J)** Cell cycle distribution of shRNA2-Lnc-FTX or sh NC transfected MHCC-LM3 Hep3B was evaluated by flow cytometry. The percentage of cells in each phase were shown;** (K-M)** Cell cycle distribution of pc-vector or pc-FTX transfected MHCC-LM3 and Hep3B was evaluated by flow cytometry. The percentage of cells in each phase were shown;** (N-O)** The apoptotic level of HCC cells was determined by flow cytometry.

High Lnc-FTX-expressing LM3 and Hep3B cell clines were selected to study the function of FTX through overexpression or knockout. We utilized MHCC-LM3 and Hep3B cell lines to construct stable cell lines. Among the various ShRNA constructs, ShRNA2-Lnc-FTX was selected for subsequent experiments due to its highest inhibition efficiency compared to ShRNA1-Lnc-FTX through ShRNA2-Lnc-FTX (Figure S1). We then established cell lines with either downregulated or overexpressed Lnc-FTX in both MHCC-LM3 and Hep3B, and confirmed the expression levels using RT-PCR (Figure S1). To evaluate the influence of Lnc-FTX on cell proliferation, Cell counting kit-8 (CCK-8) and colony formation assays were performed. We observed that cell proliferation (Figure 2A-D) and colony formation (Figure 2E-G) were considerably decreased upon Lnc-FTX knockdown, whereas both were markedly increased with Lnc-FTX overexpression. Further, we conducted flow cytometry analyses on cell cycle distribution and apoptosis. The Proliferation Index (PI) refers to the proportion of cells in the S phase and G2/M phase relative to the total number of cells. In Figure 2, the total percentage of cells in the S phase and G2/M phase in FTX knockdown cell line (shRNA2-Lnc-FTX) is lower than that of the control group (sh NC) (Figure 2H-J). The DNA content of cells in the S phase and G2/M phase exceeds the diploid amount, indicating that the cells have entered the next round of division. The reduced percentage of cells in these phases suggests that the proliferation index and proliferation activity decrease after FTX knockdown. In FTX overexpression cell line, the total percentage of cells in the S phase and G2/M phase is higher than that of the control group (Figure 2K-M), indicating that FTX overexpression increases the proliferation index and enhances proliferation activity. These results demonstrate that FTX enhances the proliferation ability of HCC cells, while FTX knockdown suppresses proliferation. Additionally, Lnc-FTX knockdown led to increased apoptosis in both MHCC-LM3 and Hep3B cells (Figure 2N), while Lnc-FTX overexpression resulted in reduced apoptosis (Figure 2O). Overall, Lnc-FTX promotes cell proliferation in HCC by accelerating the cell cycle and inhibiting cell apoptosis.

### Lnc-FTX promotes invasion and migration of HCC cells

**Figure 3:**
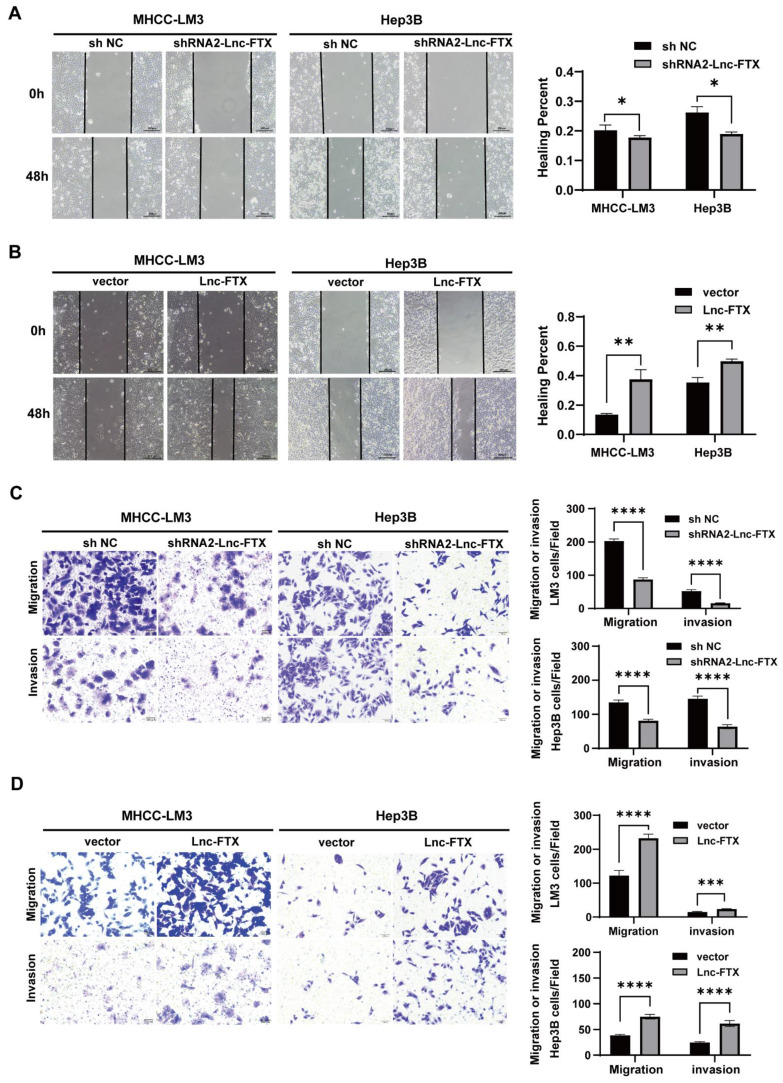
Lnc-FTX expression promotes migration and invasion in HCC. **(A, B)** The expression of Lnc-FTX was silenced treated with shRNAs and exogenously overexpressed in MHCC-LM3 and Hep3B cells. Wound-healing assays showed the increased movement capacity of HCC cells in-duced by Lnc-FTX; **(C, D)** Transwell migration and invasion assays demonstrated the effect of Lnc-FTX on the migration and invasion of HCC cells. Cells were stained using 0.1% crystal violet. The representative images and the corresponding statistical results were shown.

The wound healing assays established that Lnc-FTX knockdown significantly impaired the healing rate of cells, while upregulation of Lnc-FTX significantly accelerated it (Figure 3A, B). The Transwell assays demonstrated that, compared to their respective control cell lines, the shRNA2-FTX cell line had fewer cells passing through the Transwell chamber, whereas the cell lines with upregulated Lnc-FTX had more cells migrating through the chamber (Figure 3C, D). The upper chamber of the Transwell was coated with a layer of Matrigel for the same experimental procedure to investigate the effect of FTX on the invasion ability of HCC cells, which is consistent with the previous results (Figure 3C, D). All these findings conclude that Lnc-FTX enhances HCC cell invasion and migration.

### RNA-binding protein RBMX enhances the stability of Lnc-FTX

**Figure 4:**
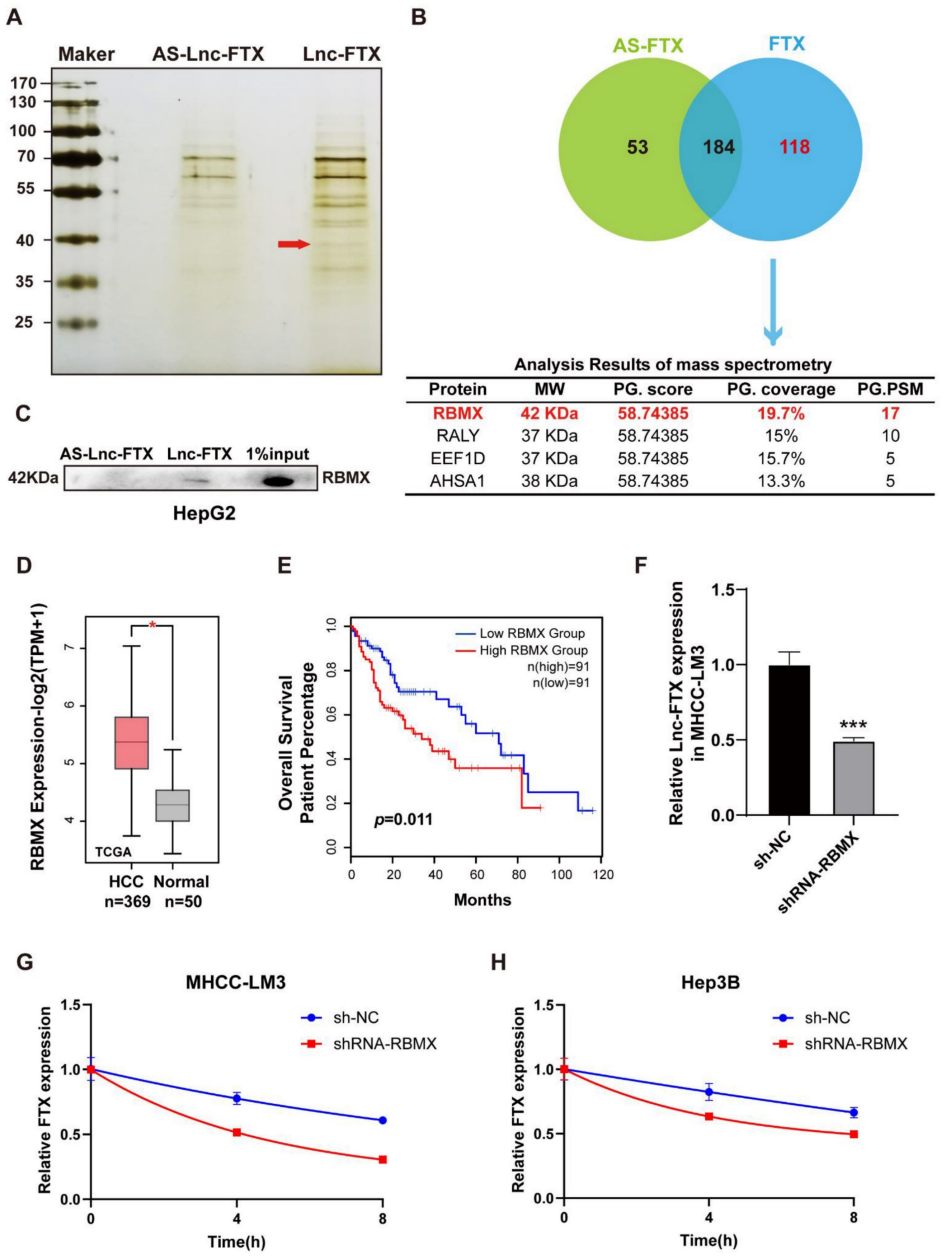
RBMX interacts with Lnc-FTX to remain its RNA stability. **(A, B)** RNA pulldown was performed using biotin-labeled Lnc-FTX probe, followed by mass spectrometry analysis; **(C)** Western Blot assay was performed to verify the interaction between Lnc-FTX and RBMX in HepG2 cells. **(D)** The mRNA level of RBMX in HCC (n = 369) and adjacent normal (n = 50) tissues was analyzed using TCGA database; **(E)** Kaplan-Meier analysis (log-rank test) of overall survival according to RBMX expression level (P < 0.01) (GEPIA); **(F)** Lnc-FTX expression level of MHCC-LM3 transfected with sh-NC or shRNA-RBMX was detected by RT-PCR analysis; **(G, H)** Measurement of Lnc-FTX RNA stability. MHCC-LM3 and Hep3B cells transfected with sh-NC or shRNA-RBMX were treated with actinomycin D to block *de novo* transcription and the levels of Lnc-FTX were assessed by RT-PCR analysis.

To explore the molecular mechanisms by which FTX regulates HCC, RNA pulldown assays (Figure 4A) were conducted to obtain candidate RBPs that directly interact with Lnc-FTX. The results revealed that 118 proteins with a molecular weight of approximately 40 kDa may specifically bind to Lnc-FTX. Among these proteins, RBMX emerged as the top candidate with the highest score (Figure 4B), thought to be crucial in functions such as alternative splicing 34 and maintaining genome stability 35, 36. Western blot analysis confirmed that RBMX existed in the pulldown protein of Lnc-FTX but not in the pulldown protein of the antisense of Lnc-FTX (Figure 4C), indicating that Lnc-FTX can directly bind to RBMX. Meanwhile, the expression level of RBMX in HCC tissues was markedly upregulated with the data from the TCGA database (Figure 4D). Moreover, the upregulated of RBMX is associated with the lower OS rates in HCC patients (Figure 4E), suggesting a potential tumor-promoting role of RBMX in HCC. RBMX knockout cells were constructed by using shRNA to explore its effect on Lnc-FTX. (Figure S2). Our findings revealed that knockdown of RBMX decreased Lnc-FTX expression (Figure 4F). Additionally, RNA stability assays demonstrated an accelerated decay rate of Lnc-FTX after RBMX knockdown (Figure 4G, H). These results collectively confirm RBMX can bind and stabilize Lnc-FTX to accelerates the progression of HCC.

### Lnc-FTX serves as a miRNA sponge miR-374-3p in HCC cells

**Figure 5:**
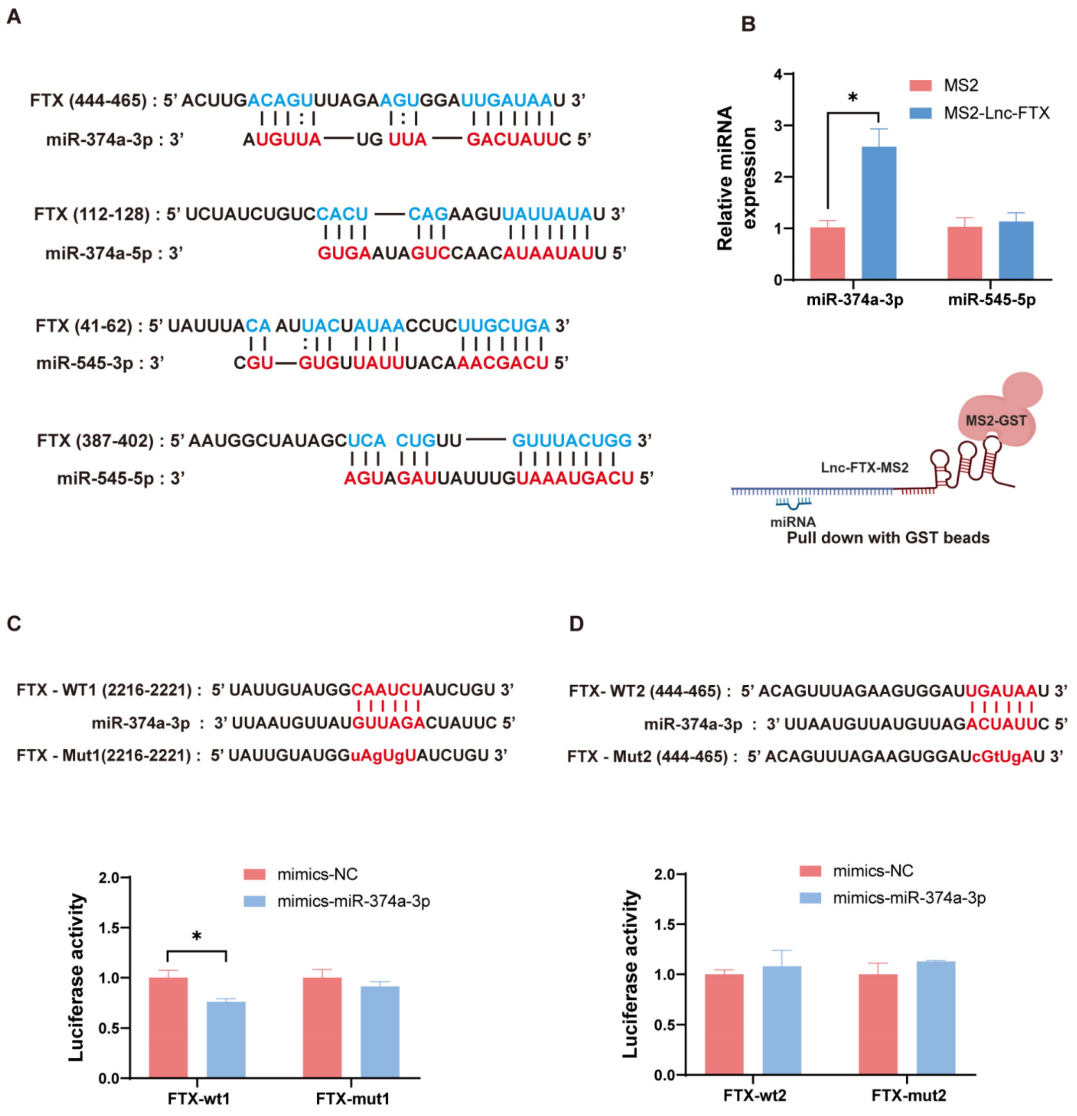
Lnc-FTX functions as a sponge of miR-374a-3p in HCC. **(A)** Schematic of predicted binding sites of miR-374a-3p, miR-374a-5p, miR-545-3p and miR-545-5p on Lnc-FTX. **(B)** RT-PCR analysis of miR-374a-3p and miR-545-5p expression in the MS2-TRAP RNA pull-down sample. **(C, D)** The predicted miR-374a-3p binding site in the FTX gene. The corresponding sequence in the mutated (mut) version is also shown. Luciferase activity of MHCC-LM3 cells transfected with NC/miR-374a-3p mimics and FTX-wt1, FTX-mut1, FTX-wt2, FTX-mut2 plasmids. Data are presented as the ratio of Firefly luciferase activity to Renilla luciferase activity.

To further investigate the downstream molecular mechanisms by which FTX promotes HCC progression, we approached the study from the competitive endogenous RNA (ceRNA) hypothesis for subsequent research. We utilized the prediction website LncBase V.2 (https://dianalab.e-ce.uth.gr) and found the binding sites between Lnc-FTX and miR-374a-3p, miR-374a-5p, miR-545-3p, and miR-545-5p. (Figure 5A). Next, we conducted a TRAP assay, and observed that miR-374a-3p was enriched in the pulldown samples of Lnc-FTX, while miR-545-5p showed no notable difference between the MS2-Lnc-FTX group and the control group (Figure 5B). Other miRNAs were undetectable. These findings indicate that Lnc-FTX can bind to miR-374a-3p. In addition, we constructed dual-luciferase gene reporter plasmids (PmiRGLO-Lnc-FTX-WT1/WT2) and their corresponding mutant plasmids (PmiRGLO-Lnc-FTX-Mut1/Mut2) for the Dual-luciferase reporter assay. We co-transfected the mentioned reporter plasmids in MHCC-LM3 cells with mimics NC and mimics miR-374a-3p, respectively. In FTX-WT1 transfected cells, the luciferase signal of the mimics miR-374a-3p group exhibited a significant decrease compared to mimics NC group. However, this effect was disappeared in FTX-mut1 transfected cells (Figure 5C). Conversely, there was no notable disparity in luciferase signal in FTX-WT2 transfected cells (Figure 5D). In conclusion, we demonstrate that Lnc-FTX binds to miR-374a-3p at FTX-WT1 site and functions as a sponge.

### Lnc-FTX upregulates HMGB1 through miR-374a-3p

**Figure 6:**
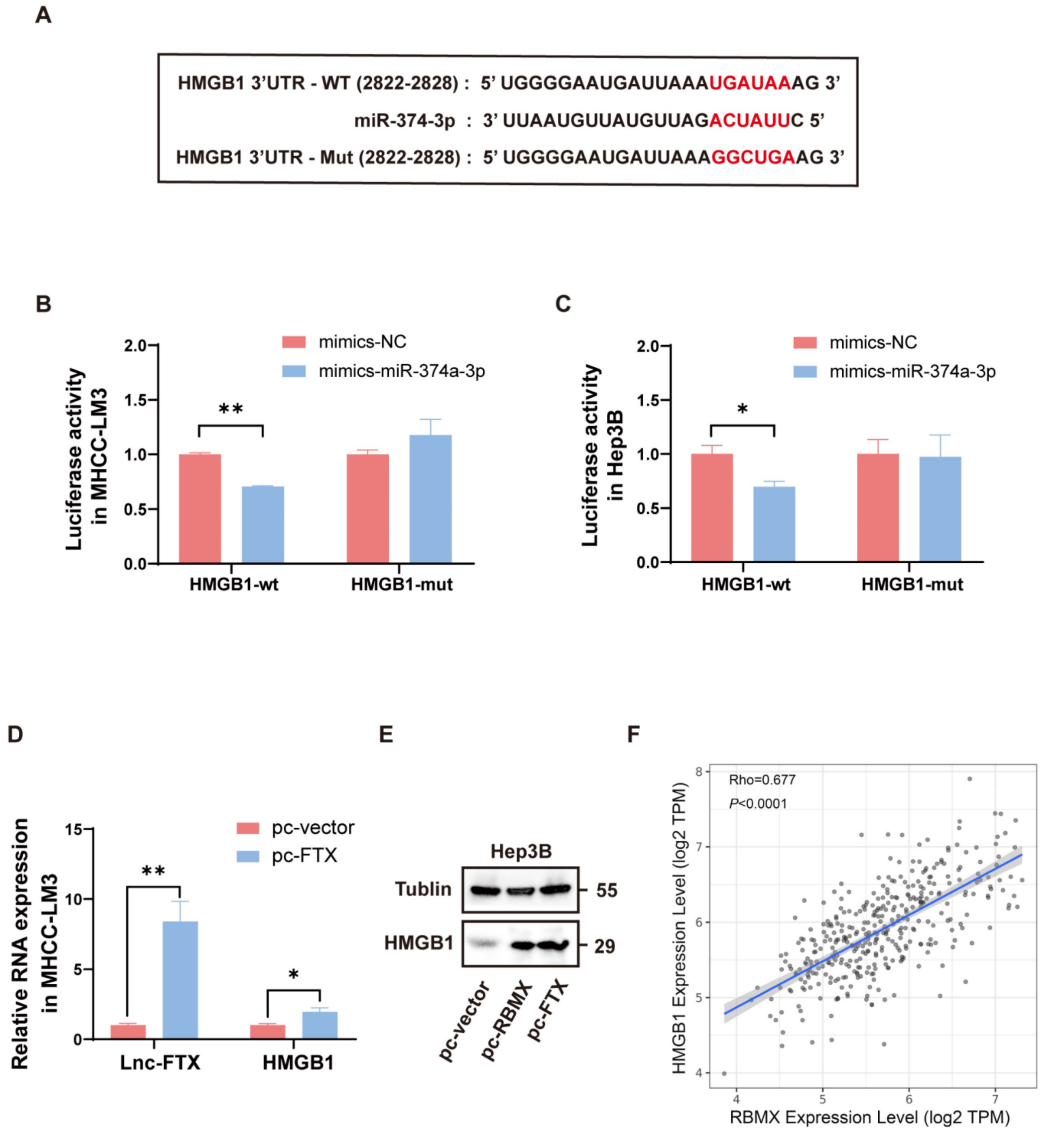
Lnc-FTX upregulates HMGB1 through miR-374a-3p.** (A)** The predicted miR-374a-3p binding site in the 3′UTR of the human HMGB1 gene. The corresponding sequence in the mutated version is also shown;** (B, C)** Luciferase activity of MHCC-LM3 and Hep3B cells transfected with NC/miR-374a-3p mimics and HMGB1-wt/mut plasmids. Data are presented as the ratio of Firefly luciferase activity to Renilla luciferase activity; **(D)** RT-PCR analysis of HMGB1 mRNA expression of MHCC-LM3 cells transfected with Lnc-FTX plasmids(pc-FTX) or control plasmids(pc-vector); **(E)** Western Blot analysis of HMGB1 expression of Hep3B cells transfected with pc-FTX/pc-RBMX or pc-vector.** (F)** The Pearson correlation analysis of the expression of RBMX and HMGB1 in TCGA (LIHC).

We predicted that there were binding sites between miR-374a-3p and HMGB1 3'UTR by analyzing online database TargetScan (http://www.targetscan.org/) (Figure 6A). Dual-luciferase reporter assay indicated that luciferase activity was notably decreased when co-transfected with HMGB1-WT and miR-374a-3p mimics, suggesting that miR-374a-3p can directly bind to the 3'UTR of HMGB1 (Figure 6B, C). Combined with the arguments above, Lnc-FTX can sponge miR-374a-3p, which can interact with its target gene HMGB1. Thus, we further investigated the effect of Lnc-FTX overexpression on the expression levels of HMGB1. We observed that HMGB1 expression was increased when Lnc-FTX was overexpressed (Figure 6D, E). These results suggest that Lnc-FTX can upregulate HMGB1 expression. Interestingly, we analyzed the TCGA database and found that there was a strong correlation between HMGB1 and RBMX expression in HCC (Figure 6F) and validated that RBMX could upregulate HMGB1 expression (Figure 6E). All these supports the existence of the Lnc-FTX/miR-374a-3p/HMGB1 regulatory axis.

## Discussion

LncRNA has been demonstrated to regulate a range of biological processes, including chromosome inactivation, nuclear transport, carcinogenesis, and disease mechanisms 37,38,39. Given the insidious and recurrent nature of cancer, it poses a significant threat to human health, highlighting the importance of identifying effective therapeutic targets 40,41. Recently, lncRNA has gained considerable attention for its roles in cancer, with many, such as HOTAIR, HULC, HOTTIP, and NEAT1, identified as oncogenes 42. Overall, this study confirms that the overexpression of Lnc-FTX in HCC is correlated with dismal prognosis, providing valuable guidance for clinical risk assessment. Additionally, the promotion of proliferation, invasion, and migration by Lnc-FTX in HCC further supports its role as an oncogene.

Lnc-FTX functions as an oncogene across various cancers, facilitating the malignant behaviors of cancer cells. A study on lung adenocarcinoma demonstrated that Lnc-FTX is upregulated in tumor tissues and accelerates malignant characteristics of Lung adenocarcinoma by activating the miR-335-5p/NUCB2 pathway 43. Research by Kui Zhao *et al.* revealed that Lnc-FTX is positively regulates miR-192-5p to target EIF5A2, thereby enhancing cancer cell proliferation ability in colorectal cancer cells 44. Consistent with these observations, our research identified that Lnc-FTX expression level was markedly elevated in HCC tissues with the data from GSE77314 and TCGA. Clinical data from a cohort of 27 HCC patients (cohort 1) showed that high Lnc-FTX expression is strongly associated with advanced TNM classification and reduced OS, indicating a correlation between high Lnc-FTX levels and poor prognosis. Nonetheless, in cohort 1, the expression of lnc-FTX did not show a significant difference between cancerous and adjacent non-cancerous tissues. It may be attributed to: (1) Tumor heterogeneity: Variability in tumor cells across individuals. (2) Sample size impact: A larger clinical sample size will help more accurately reflect the expression levels of Lnc-FTX. (3) TNM staging relevance: Lnc-FTX levels correlate with TNM staging, with lower Lnc-FTX expression observed in patients with lower TNM stages (Stage I). Thus, the higher number of patients in lower TNM stages in our cohort likely explains the increased prevalence of lower Lnc-FTX expression in tumor tissues.

LncRNAs exert their influence through a variety of mechanisms: decoys, scaffolds, guidance, stabilization, ceRNA or "RNA sponge" 45. It is summarized in two ways: (1) direct binding to regulatory regions of genes, such as promoters, enhancers and transposons, to recruit the relevant protein complexes, thereby affecting chromatin remodeling and transcription 45. (2) Binding to intermediate regulators, including RBPs or microRNAs 46, which is the mainstream molecular mechanism of LncRNA regulation in tumor progression 47.

RNA-protein interactions participate in many essential cellular functions, such as splicing, polyadenylation, stability, and translation 48. RBMX, commonly referred to as hnRNP G, is a widely expressed nuclear RBP. It plays a role in normal developmental processes, such as brain development, and is associated with the regulation of multiple diseases, including cancer (activation in liver cancer), systemic lupus erythematosus, and viral infections 49. Its primary biological functions include involving in chromatin modification, splicing and alternative splicing regulation 50, transcriptional control, and maintenance of genomic stability 51. RBMX is vital to the progression of HCC, although research into the underlying mechanisms is still limited. Recent research indicates that RBMX can bind with SOCS5 to co-stimulate the SREBP1 promoter, and lipid accumulation induced by SREBP1 may promote the metastasis of steatotic HCC 52. Another study on HCC shows that RBMX specifically binds to and stabilizes Lnc-BLACAT1, thereby promoting malignant phenotypes of HCC and resistance to chemotherapeutic drugs 53. Our findings are in accord with these studies indicating that RBMX binds to Lnc-FTX and enhances Lnc-FTX stability and suggesting that RBMX is of clinical significance to be one of potential therapeutic targets for HCC.

Among the multiple models of lncRNA-RNA interactions, the ceRNA hypothesis has garnered significant attention. This hypothesis posits that LncRNAs, miRNAs, and mRNAs engage in a complex network of interactions. LncRNAs serves as ceRNAs or natural microRNA sponges, which regulate mRNAs by competitively inhibiting miRNAs in the presence of microRNA response elements 54. To further identify the downstream targets of Lnc-FTX, LncBase Predicted V2 was used to predict the associated candidate microRNAs, with miR-374a-3p confirmed as one of the potential downstream targets. HMGB1, a well-known oncogene, plays a critical role in regulating breast cancer, gastrointestinal stromal tumor and melanoma 55. A significant amount of evidence demonstrates that HMGB1 is engaged in tumor cell malignant biological processes through signaling pathways such as NF-κB, JAK/STAT and PI3K/AKT 56. This study predicted and confirmed a miR-374a-3p binding site in HMGB1. Up-regulation of Lnc-FTX led to elevated HMGB1 expression. Coupled with the previous finding, Lnc-FTX regulates HMGB1 through miR-374a-3p. The Lnc-FTX/miRNA-374a-3p/HMGB1 axis regulation for HCC also brings new perspectives for clinical treatment.

This study innovatively explored the interaction between Lnc-FTX and HMGB1 and discovered a strong association between the expression of RBMX and HMGB1. Overexpression of RBMX in HCC cells led to a significant upregulation of HMGB1, indicating that RBMX can enhance HMGB1 expression. In summary, the establishment of FTX/miR-374a-3p/HMGB1 axis via the RBMX stabilizing the FTX provides new directions for future research into the molecular mechanisms of HCC.

## Supplementary Material

Supplementary figures and tables.

## Figures and Tables

**Table 1:** Association of Lnc-FTX with clinicopathologic characteristics of patients with hepatocellular carcinoma.

Variable		Lnc-FTX expression level		p
		High (n=11)	Low (n=16)	
Age group	≤55	5	9	0.704
	>55	6	7	
Sex	Male	9	15	0.549
	Female	2	1	
Edmondson grade	II	7	12	0.452
	II-Ⅲ	3	4	
	Ⅲ	1	0	
TNM classification	I	4	9	0.027*
	II	4	0	
	Ⅲ	1	6	
	Ⅳ	2	1	
Tumor size (cm)	≤5	6	7	0.704
	>5	5	9	
Tumors	Multiple	5	7	>0.999
	Solitary	6	9	
BCLC stage	0+A	1	1	>0.999
	B+C	11	15	
Vascular invasion	Yes	1	1	>0.999
	No	10	15	
Capsular invasion	Yes	4	5	>0.999
	No	7	11	
Cirrhosis, No. (%)	Yes	9	7	0.109
	No	2	9	

* TNM = tumor node metastasis; BCLC = Barcelona Clinic liver cancer. The low expression group included 16 patients whose Lnc-FTX expression in tumor tissues was < or equal to the normal tissues. The high expression group included 11 patients whose Lnc-FTX expression in tumor tissues was > the normal tissues. The Pearson χ2 test and Chi-Squared Test were used for the analysis of the correlation between Lnc-FTX expression levels and clinical characteristics. *P < 0.05
