# Supplementary material for: LncRNA FTX accelerates the progression of hepatocellular carcinoma by FTX/miR-374a-3p/HMGB1 pathway
Source: Int J Med Sci. 2025 Feb 18;22(6):1363–74. doi: 10.7150/ijms.106867 (PMC11898846; doi:10.7150/ijms.106867)
Supplement: Supplementary file 1 — Supplementary figures and tables. [file ijmsv22p1363s1.zip › supplementary material/Supplemental Materials.pdf]

LncRNA FTX accelerates the progression of hepatocellular carcinoma by FTX/miR-374a-3p/HMGB1 pathway

Min Zhang, Songman Yu, Shang Gao, Haiyan Bu, Lihua Duan, Yan Huang

Table of contents

Supplementary Material and Methods Doc S1 ………………………………2

Supplemental Figure1…………………………………………………………5

Supplemental Figure2…………………………………………………………6

Supplemental Table1…………………………………………………………….7

Supplemental Table1…………………………………………………………….8

Supplementary Material and Methods Doc S1

RNA extraction and RT-PCR

The total RNA Kit (Omega, USA) and the miRNeasy FFPE RNA kit (QIAGEN, German) were used for RNA extraction. The PrimeScript RT reagent kit (Takara, Japan) and TB Green Premix Ex Taq^{TM} II (Takara, Japan) were used for reverse transcription and real-time PCR with Real-time fluorescence quantitative PCR instrument (BIO-RAD, USA). The transcriptional expression was determined using the value of $2^{-\Delta\Delta CT}$.

Western blot

RIPA Lysis Buffer (Beyotime, China) supplemented with a 100x Protease Inhibitor Cocktail (Bimake, USA) was used to obtain Cell lysates. Quantification of Protein Concentration Using a BCA Protein Assay Kit (Thermo, USA). Primary antibodies against HMGB1 (ab18256, Abcam, USA), RBMX (ab190352, Abcam, USA) and β-Tublin (A12289, ABclonal, China) were used. Following incubation with secondary antibodies (FSM0075, Fushen, China), membranes were developed with western ECL Substrate (1705062, BIO-RAD, USA) and the blots were detected with the enhanced chemiluminescence system (BIO-RAD, USA).

Plasmids and transfection

The expression vector pcDNA3.1 (+) containing full-length Lnc-FTX was purchased from Sangon Biotech (Shanghai, China) and transfected into MHCC-LM3 and Hep3B cells with Lipofectamine 2000 reagent (Invitrogen, USA) according to the manufacturer's instructions. We used 6.25 ug/ml and 100 ug/ml Geneticin™ (Thermo Fisher Scientific, USA) for a two-week selection of stable cell line respectively. We inserted NC shRNA or Lnc-FTX shRNA1-shRNA5 (Table S2) into the lentiviral vector PLV (Biosettia, USA). We transfected them using 10μg/ml polybrene (Beyotime, Shanghai, China), and then selected stable cell lines with 2.5μg/ml puromycin (Yeasen, Shanghai, China). Cells were transiently transfected with a

siRNA NC or siRNAs targeting Lnc-FTX by using Lipofectamine 8000 reagent (Beyotime, Shanghai, China). The efficiency of Lnc-FTX overexpression or knockdown was verified by qRT-PCR (Figure S1).

Cell viability and colony formation assays

Cell proliferation was examined with the Cell Counting Kit 8 (CCK8) (Ecotop, China) according to the manufacturer's protocols. Cells were seeded in 96-well plates at a density of 5×103 cells/well and cultured for 4 days. CCK8 reagent was added to each well and incubated at 37°C for 2 h. The absorbance of each well was detected at 450 nm using a PerkinElmer spectrophotometer (Shanghai, China). For the colony formation assay, cells were plated in 6-well plates (1000cells/well) and cultured for 2 weeks. Colonies were fixed with 4% paraformaldehyde and stained with 0.1% crystal violet for 30 min.

Flow cytometry analysis of cell cycle and apoptosis

Cell cycle assay was performed with the cell cycle staining kit (Multi sciences, China). Cells plated in 6-well plates were harvested after 24h starvation and adjusted to a concentration of 2×105/ml. Cells were fixed overnight in 75% ethanol at 4 °C, then washed in PBS twice, incubated with the PI/RNase A (9:1) staining solution mixture for 30 minutes away from light. Flow cytometry was performed using a BD FACSCanto II Flow Cytometer (BD Biosciences, USA). For the apoptosis assay, cells were harvested and incubated with Annexin V FITC and PI according to the manufacturer’s instructions (4A Biotech, China). The stained cells were detected by flow cytometry.

Wound-healing assay

The gap between cells were created by a sterile pipette tip 200 μl. Cells were cultured in serum-free medium, and images were captured at 0h, 24h and 48h by an inverted microscope

(CTR5000, LEICA, German). The migration rate was calculated by comparing the area at different time points to that at 0h.

Transwell assay

Transwell assay was conducted using the 8 μm pore-size transwell plates (Corning, USA). Specifically, cells were seeded in upper chambers at a density of 5 × 104 cells/well with FBS-free medium for migration, or 1:8 diluted Matrigel (Corning, USA) for invasion. The lower chambers were filled with medium containing 10% FBS. After 36 h, cells were fixed with 4% paraformaldehyde and stained with a 0.1% crystal violet solution (Beyotime, USA) for 30 min.

Supplementary figure 1:

Validation of Knockdown and Overexpression Efficiency of Lnc-FTX

(A) Detection of Lnc-FTX expression in MHCC-LM3 cell line 48 hours after transfection with shRNA (FTX-sh1~5) compared to ShNC-transfected cells by qRT-PCR. (B) Detection of Lnc-FTX expression 48 hours after transfection with pcDNA3.1 and pc-FTX plasmids in Hep3B and MHCC-LM3 cells by qRT-PCR.

Figure S1

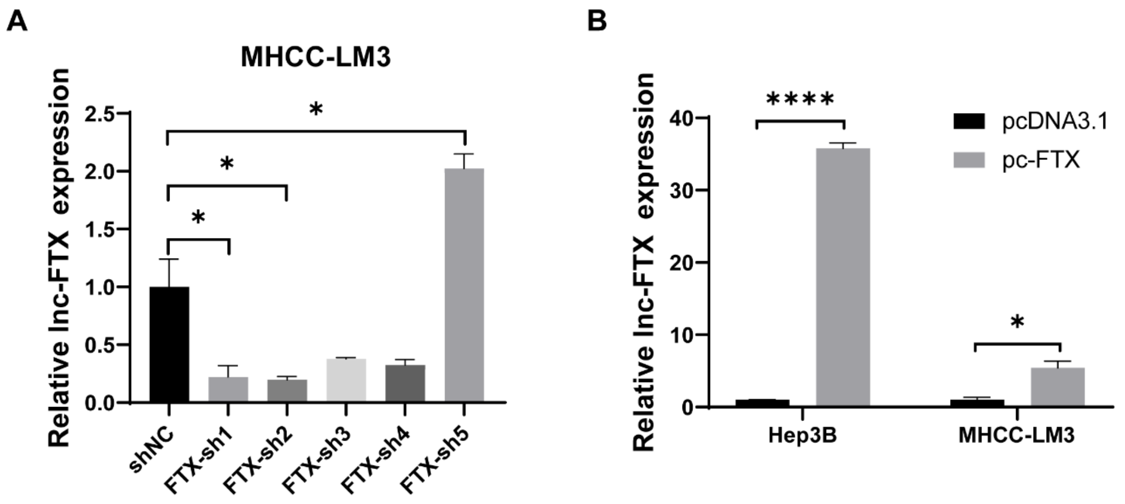

Supplementary figure 2:

Validation of Knockdown and Overexpression Efficiency of RBMX

(A) Detection of RBMX expression in MHCC-LM3 cell line 48 hours after transfection with shRNA (RBMX-sh1~2) compared to ShNC-transfected cells by Western Blot analysis. (B) Detection of RBMX expression 48 hours after transfection with pcDNA3.1 and pc-RBMX plasmids in MHCC-LM3 cells by Western Blot analysis. (C) Representative Western blot image showing Lnc-FTX knockdown or overexpression

Figure S2

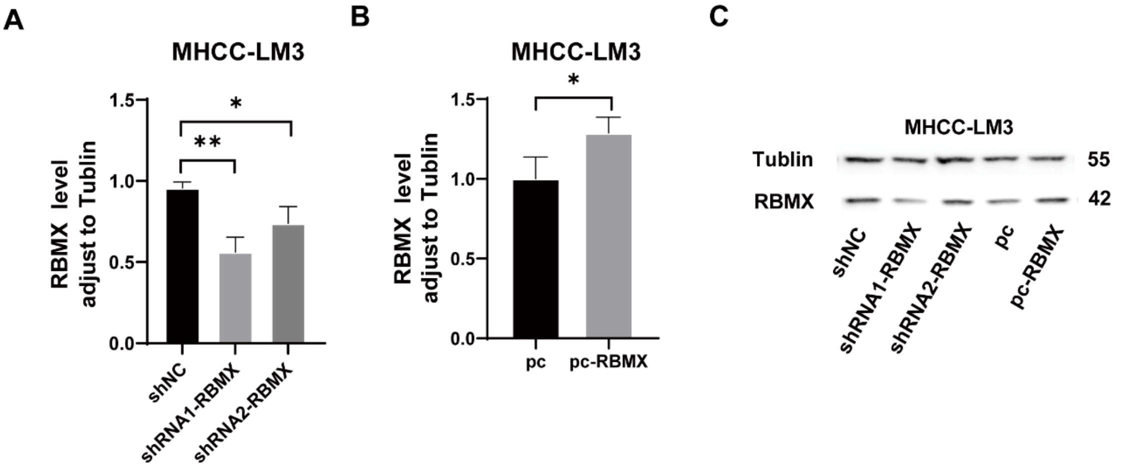

Supplementary table 1:

Clinical and pathologic characteristics of patients with hepatocellular

**characteristics**	**Value**
Age, mean (SD)	52.3 (12.8)
Sex(male/female)	24/3
Liver cirrhosis, no. (%)	16 (59.2)
Tumor size, mean (SD), mm	7.0 (5.6)
Tumor size ≥50 mm, no. (%)	13 (48.1)
Tumor number (solitary/multiple)	16/11
Edmondson-Steiner grade(II/II-III/III)	19/7/1
TNM stage(II/II-III/III)	13/4/7/3
BCLC stage, A/B/C	2/2/23
Capsular invasion, no. (%)	2 (7.4)
Vascular invasion, no. (%)	9 (33.3)

*BCLC = Barcelona Clinic liver cancer; TNM = tumor node metastasis.

Supplementary table 2:

Primers, shRNA and siRNA used in this study

primer	Sequence (5’ to 3’)
GAPDH-homo (F)	AGCCACATCGCTCAGACAC
GAPDH-homo (R)	AATCCGTTGACTCCGACCT
Lnc-FTX-homo (F)	GGCGAGGATGAATTACGG
Lnc-FTX-homo (R)	CAAGGCTTCCCAACTGC
HMGB1-homo (F)	CCGTCTGTGCCTTCTCAT
HMGB1-homo (R)	CGTTCACGGTGGTCTCC
RBMX-homo (F)	GCCCAGCAGACGCTAAGGA
RBMX-homo (R)	TGGTTTGGTGGCTTGTTCC
T7-Ftx(F)	TAATACGACTCACTATAGGGGGAAGCACACTGGC
T7-Ftx (R)	GGGAAATAAGTTTATTAC
T7-AS-Ftx(F)	GGAAGCACACTGCG'
T7-AS-Ftx(R)	TAATACGACTCACTATAGGGGGAAATAAGTTTATTA<br>C
h-ShNC-(F)	GATCCGTTCTCCGAACGTGTCACGTTTTTCAAGAGAAA<br>ACGTGACACGTTCGGAGAATTTTTTG
h-ShNC-(R)	AATTCAAAAAATTCTCCGAACGTGTCACGTTTTCTCTT<br>GAAAAACGTGACACGTTCGGAGAACG
h-FTX (332)-sh1 (F)	GATCCGGCATCCTGCACCTAGTTATCATTCAAGAGATG<br>ATAACTAGGTGCAGGATGCTTTTTTG
h-FTX (332)-sh1 (R)	AATTCAAAAAAGCATCCTGCACCTAGTTATCATCTCTT<br>GAATGATAACTAGGTGCAGGATGCCG

h-FTX (432)-sh2 (F)	GATCCGGCCTGTTACTCATACTGATGCTTCAAGAGAGC ATCAGTATGAGTAACAGGCTTTTTTG
h-FTX (432)-sh2 (R)	AATTCAAAAAAGCCTGTTACTCATACTGATGCTCTCTT GAAGCATCAGTATGAGTAACAGGCCG
h-FTX (526)-sh3 (F)	GATCCGGCTGGATTGTGAAGGATATTTTTCAAGAGAA AATATCCTTCACAATCCAGCTTTTTTG
h-FTX (526)-sh3 (R)	AATTCAAAAAAGCTGGATTGTGAAGGATATTTTCTCTT GAAAAATATCCTTCACAATCCAGCCG
h-FTX (1459)-sh4 (F)	GATCCGGGACAAATCCCATTTGAACATTCAAGAGAT GTTCAAATGGGATTTGTCCCTTTTTTG
h-FTX (1459)-sh4 (R)	AATTCAAAAAAGGGACAAATCCCATTTGAACATCTCT TGAATGTTCAAATGGGATTTGTCCCCG
h-FTX (2174)-sh5 (F)	GATCCGGCTGAAACTTCAGTACTTAAGTTCAAGAGAC TTAAGTACTGAAGTTTCAGCTTTTTTG
h-FTX (2174)-sh5 (R)	AATTCAAAAAAGCTGAAACTTCAGTACTTAAGTCTCTT GAACTTAAGTACTGAAGTTTCAGCCG
h-RBMX-shRNA (F)	GATCCGGAGAAACGAATAAGTCAAGATTCAAGAGATC TTGACTTATTCGTTTCTC TTTTTT G
h-RBMX-shRNA (R)	AATTCAAAAAA GAGAAACGAATAAGTCAAGATCTCTTGAATCTTGACTT ATTCGTTTCTCCG
si-FTX-Homo-423 (F)	GCCUGUUACUCAUACUGAUGCTdT
si-FTX-Homo-423 (R)	GCAUCAGUAUGAGUAACAGGCTdT
Si-NC (F)	Purchase from RiboBio (Sequence confidentiality)
Si-NC (R)	Purchase from RiboBio (Sequence confidentiality)
HsnRNA U6 primer	Purchase from FulenGen Cat.HmiRQP9001(Sequence confidentiality)

Has-miR-374a-3p primer	Purchase from FulenGen Cat.HmiRQP0462(Sequence confidentiality)
Has-miR-545a-5p Primer	Purchase from FulenGen Cat.HmiRQP0625(Sequence confidentiality)
